# Inhibitory effect of Ubenimex combined with fluorouracil on multiple drug resistance and P‐glycoprotein expression level in non‐small lung cancer

**DOI:** 10.1111/jcmm.15875

**Published:** 2020-09-17

**Authors:** Jun Wan, Xie‐an Ling, Jian Wang, Guang‐gui Ding, Xi Wang

**Affiliations:** ^1^ Department of Thoracic Surgery Shenzhen People’s Hospital The Second Clinical Medical College of Jinan University The First Affiliated Hospital of South University of Science and Technology Shenzhen China; ^2^ Department of Critical Care Medicine Shenzhen People’s Hospital The Second Clinical Medical College of Jinan University The First Affiliated Hospital of South University of Science and Technology Shenzhen China

**Keywords:** 5FU, lung cancer, multiple drug resistance, P‐gp, Ubenimex

## Abstract

Tumour drug resistance is one of the most urgent issues faced by anti‐tumour therapies. P‐glycoprotein (P‐gp) has been reported to be correlated with drug resistance. In this study, we aimed to study the synergistic effect of fluorouracil (5FU) and Ubenimex (UBE) on drug resistance in lung cancer. In this study, the tumour inhibitory role of 5FU and UBE was assessed in nude mice bearing A549 or A549/ADR. Real‐time polymerase chain reaction, Western blot and immunohistochemical were performed to analyse the mRNA and protein expression of P‐gp. TUNEL assay was used to evaluate the apoptosis of A549/ADR cells under 5FU and UBE treatment. MTT assay was performed to calculate the IC50 value of 5FU and UBE in A549 or A549/ADR. Combined administration of 5FU and UBE significantly inhibited the tumour growth of multidrug‐resistant cell lines A549/ADR in nude mice by down‐regulating the mRNA and protein expression of P‐gp. The apoptosis of A549/ADR was remarkably elevated in nude mice treated with 5FU and UBE. The IC50 value of 5FU and UBE was dramatically declined in A549/ADR cells compared with that of 5FU or UBE alone. Combined treatment of 5FU and UBE remarkably enhanced the apoptosis of A549/ADR cells by enhancing the intracellular accumulation of the drugs. The results of this study demonstrated that UBE combined with fluorouracil attenuated multiple drug resistance and inhibited the expression of P‐gp in lung cancer.

## 1 INTRODUCTION

As a major contributor of global mortality caused by cancers, lung cancer is currently mainly treated by surgical resections, including the dissection of mediastinal lymph nodes by lobectomy operations, especially for patients suffering from non‐metastatic non‐small cell lung cancer.^1^ Nevertheless, in many patients suffering from non‐metastatic non‐small cell lung cancer, surgical operations are out of consideration because these patients have poor pulmonary functions and a high risk of cardiovascular diseases and other comorbidities. For these patients, chemotherapy and adjuvant therapies are used as the first‐line treatments.^2^

Ubenimex (UBE), also known as bestatin, is a drug with immune‐modulatory and anti‐tumour activities. At present, it is applied in the treatment of patients with non‐lymphocytic leukaemia.^3^ The delivery of UBE into the body can be enhanced by several transporters.^4^ For example, the absorption of UBE in the intestinal tract can by enhanced by both PEPT2 and PEPT1. In addition, the excretion of UBE, 80% of which is excreted through the urine, from the kidney can be facilitated by OATs and OCTs.^5^, ^6^ Moreover, the therapeutic efficacy of UBE can also be impacted by a wide range of drug transporters. In one study, UBE was found to be preferably delivered into tumour cells overexpressing PEPT1, suggesting that PEPT1 plays an essential role in the cellular uptake of UBE.^7^ In addition, the combination of UBE with chemotherapy reduces the tumour size while prolonging the survival of cancer patients suffering from squamous cell lung cancer.^8^, ^9^ As a result, it is suspected that UBE can play an anti‐tumour role in the treatment of squamous cell cancer and non‐small cell lung cancer by inducing the apoptosis of cancer cells.

Fluorouracil, also known as 5FU, is a derivative of certain drugs widely applied in the chemotherapy of cancer treatments. The anti‐tumour effect of 5FU is mediated through its ability to inhibit the activation of thymidylate synthase. Upon its entry into cells, 5FU can be metabolized in the cytoplasm by a wide range of metabolic enzymes, such as thymidine phosphorylase, orotate phosphoribosyl transferase and dihydropyrimidine dehydrogenase, into dihydrofluorouracil, which is inactive, as well as other metabolites with the ability to block the function of thymidylate synthase in the synthesis of RNA and DNA required in cell replication, thus disrupting the proliferation and growth of cancer cells.^10^

In a cancer cell line KB‐8‐5, the treatment with 5FU can reduce the expression of P‐glycoprotein (P‐gp) to render cancer cells more sensitive to the toxicity of Taxol. At the same time, an elevated level of CAF expression in tumour tissues also elevates the level of collagen expression. On the other hand, the treatment of tumour with 5FU reduces the multiple drug resistance (MDR) of tumour cells by reducing the level of P‐gp expression, an important factor implicated in the onset of MDR.

The treatment with UBE can inhibit the levels of expression of proteins responsible for the induction of MDR, such as MRP1 and P‐gp, leading to an elevated level of accumulation of chemotherapeutic drugs in cancer cells. In this study, we studied the synergistic effect of 5FU and UBE in the control of MDR in lung cancer both in vitro and in vivo.

## 2 MATERIALS AND METHOD

### 2.1 Animals

In this study, a total of 28 nude C57BL/6 mice were purchased from the experimental animal centre of our institution and adapted for 7 days in a SPF grade animal facility before they were randomly divided into the following 4 groups: 1. SHAM group (N = 7); 2. 5FU group (N = 7); 3. UBE group (N = 7); and 4. 5FU + UBE group (N = 7). The study on tumour growth inhibition was subsequently carried out by xenografting the nude mice with A549 (a lung carcinoma tissue‐derived human epithelial cell line sensitive to chemotherapy) or A549/ADR cells (A549 cell line which was induced of MDR), followed by a subsequent treatment with 5FU and UBE monotherapies or the combination of 5FU and UBE for 12 successive days. The tumour volumes were calculated as (length × width^2^)/2 from measurements taken every other day. The doses of 5FU and UBE used in this experiment were 25 and 10 mg/kg. Institutional ethical committee has approved the protocol of this study.

### 2.2 Cell culture and treatment

Human A549 cells were acquired from the Shanghai Cell Bank of the Chinese Academy of Sciences (Shanghai, China) and were both cultured in a standard RPMI‐1640 medium added with 0.1 mg/mL of streptomycin, 100 Unit/mL of penicillin and 10% (v/v) of foetal bovine serum. The cells were maintained under saturated humidity and an atmosphere of 95% air and 5% CO_2_ in a 37°C incubator. To screen out an A549/ADR cell line, the A549 cells were subjected to continuous antibiotic selection in a standard RPMI‐1640 medium added with 0.1 mg/mL of streptomycin, 100 Unit/mL of penicillin, 10% (v/v) of foetal bovine serum and 1 mg/mL of doxorubicin. Usually, the cells were screened for 2 weeks before an MDR clone could be established. After the successful establishment of the A549/ADR cell line, both A549 cells and A549/ADR cells were divided into 4 groups, that is (a) UNTREATED group; (b) 5FU group; (c) UBE group; and (d) 5FU + UBE group. The cells in various groups were treated with corresponding treatments. In groups 2‐4, the concentrations of 5FU and UBE were 0.00 and 0.00 mg/mL, respectively. After the cells were treated for 72 hours, they were harvested and used for subsequent assays.

### 2.3 Accumulation of UBE

Both A549/ADR cells and A549 cells were treated in the presence or absence of 10 mmol/L of 5FU for 24 hours before the level of UBE accumulation in the cells was measured by utilizing liquid chromatography‐mass spectrometry assay. In brief, the treated cells were collected by trypsinization and re‐suspended in 4°C phosphate‐buffered saline with the concentration of cells adjusted to 2 × 10^6^ cells/mL. After being incubated for 10 minutes in a 37°C incubator, 5 mmol/L of UBE was added into the suspension of cells either without or with 20 mmol/L of verapamil. In the next step, the cells were further cultured for 30 minutes in the 37°C incubator before they were collected via 10 minutes of centrifugation at 1500*g* and 4°C for subsequent analyses.

### 2.4 RNA isolation and real‐time PCR

The total RNA content in cultured cell samples and collected tissue samples was extracted by utilizing an RNA ISO Plus assay kit (Takara) in accordance with the instructions provided by the manufacturer. In the next step, the extracted total RNA was reversely transcribed to cDNA by utilizing an Omniscript RNA Reverse Transcription Assay Kit (Qiagen). Then, the obtained cDNA was subjected to real‐time polymerase chain reaction (PCR) carried out on a 7500 real‐time PCR instrument (Applied Biosystems) in conjunction with a SYBR Green Master Mix (Thermo Fisher Scientific) in accordance with the instructions provided by the manufacturer. Finally, the relative expression of P‐gp mRNA was calculated based on the threshold value of the real‐time PCR amplification of P‐gp and normalized to the expression of housekeeping gene GAPDH. The forward and reverse primers for P‐gp mRNA was 5′‐GCTGTCAAGGAAGCCAATGCCT‐3′ and 5′‐TGCAATGGCGATCCTCTGCTTC‐3′. And the forward and reverse primers for GAPDH was 5′‐GTCTCCTCTGACTTCAACAGCG‐3′ and 5′‐ACCACCCTGTTGCTGTAGCCAA‐3′.

### 2.5 Western blot analysis

Cultured cell samples and collected tissue samples were first lysed in a passive lysis reagent (Promega) added with 10 μg/mL of phosphatase inhibitor and 10 μg/mL of protease inhibitor. Then, each lysate sample was subjected to the analysis by a BCA Protein Assay (Pierce, Thermo Fisher Scientific) to determine its protein concentration. The protein samples (50 μg of protein in each sample) were then loaded onto a 10% SDS‐PAGE gel and resolved under electrophoresis. The resolved proteins were blotted onto PVDF membranes (Amersham), which were then blocked with 10% skim milk, incubated for 24 hours with anti‐P‐gp primary antibodies (cat.no. ab170940; dilution 1:5000; Abcam), anti‐PIM‐3 primary antibodies (cat.no. ab198842; dilution 1:1000; Abcam), anti‐p‐PI3K primary antibodies (cat.no. ab135253; dilution 1:2000; Abcam) and anti‐p‐AKT primary antibodies (cat.no. ab38449; dilution 1:1000; Abcam) at 4°C and then further incubated for 2 hours with corresponding HRP‐conjugated secondary antibodies at room temperature (Amersham). Finally, the PVDF membranes were developed in an ECL reagent (Thermo Fisher Scientific) and visualized underneath an X‐ray instrument to determine the relative protein expression of P‐gp using β‐actin as the internal reference.

### 2.6 Immunohistochemistry assay

The collected tissue samples were embedded in paraffin, de‐paraffinized with xylene, re‐hydrated with gradient alcohol, treated with 3% H_2_O_2_ for 10 minutes to block non‐specific binding and then incubated at 4°C overnight with rabbit anti‐mouse P‐gp primary antibodies (Santa Cruz Biotech). After washing, the samples were further incubated at room temperature for 2 hours with a PE‐tagged goat anti‐rabbit secondary antibody (Santa Cruz Biotech) and counterstained with DAPI (Sigma Aldrich) before the image of each sample was acquired utilizing an IX‐71 inverted fluorescence microscope (Olympus) to determine the positive expression of P‐gp proteins.

### 2.7 TUNEL assay

In this study, a TUNEL assay kit (Thermo Fisher Scientific) was used to determine the status of cell apoptosis of each sample in accordance with the instructions provided by the manufacturer.

### 2.8 Statistical analysis

All data were statistically analysed utilizing SPSS 21.0 statistical software (SPSS). The differences among different groups were compared using the Student's *t* tests and one‐way analysis of variance. All experimental results were presented as mean ± standard deviations. A *P*‐value of .05 was set as the threshold for statistical significance.

## 3 RESULTS

### 3.1 Combined administration of 5FU and UBE overcame the Multidrug Resistance (MDR) of A549/ADR in nude mice

**FIGURE 1:**
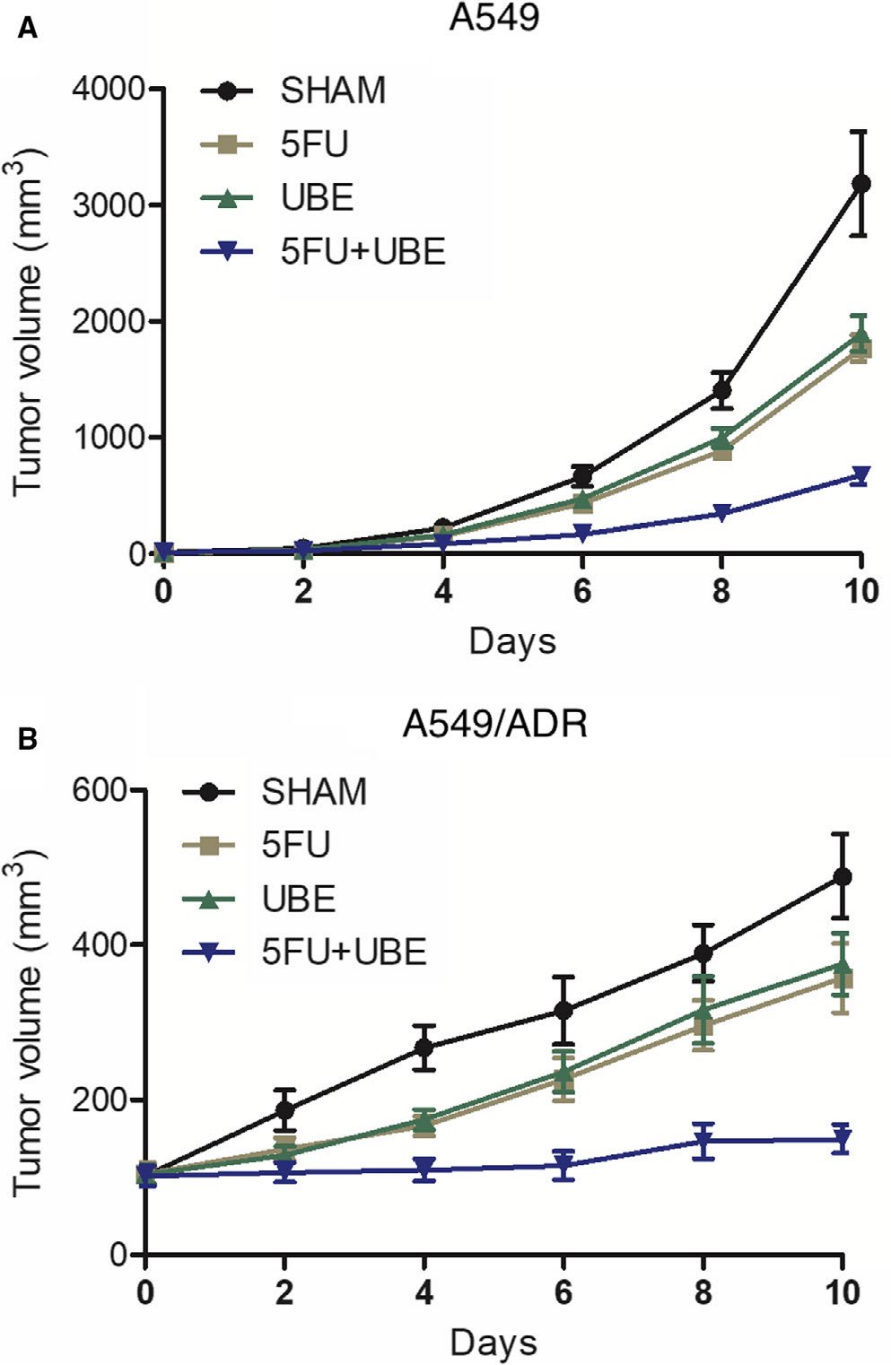
The growth of A549/ADR in nude mice was significantly inhibited by the combined administration of 5FU and UBE. A, Growth inhibition of A549 tumour in nude mice was more significantly promoted by 5FU + UBE in comparison with 5FU or UBE alone. B, Growth inhibition of A549/ADR tumour in nude mice was more significantly promoted by 5FU + UBE in comparison with 5FU or UBE alone

A549/ADR was an established MDR cell line derived from A549, a well‐known immortalized cell line of myelogenous leukaemia. The tumour size was measured every other day to evaluate the tumour inhibitory effects of the drugs. Compared with 5FU and UBE mono‐therapies, tumour growth was more significantly repressed by the combined administration of 5FU and UBE in both A549 (Figure 1A) and A549/ADR (Figure 1B) cells, indicating that the combined administration of 5FU and UBE effectively overcame the MDR and promoted the drug efficacy.

### 3.2 Combined administration of 5FU and UBE inhibited the expression of P‐gp in nude mice bearing A549/ADR

**FIGURE 2:**
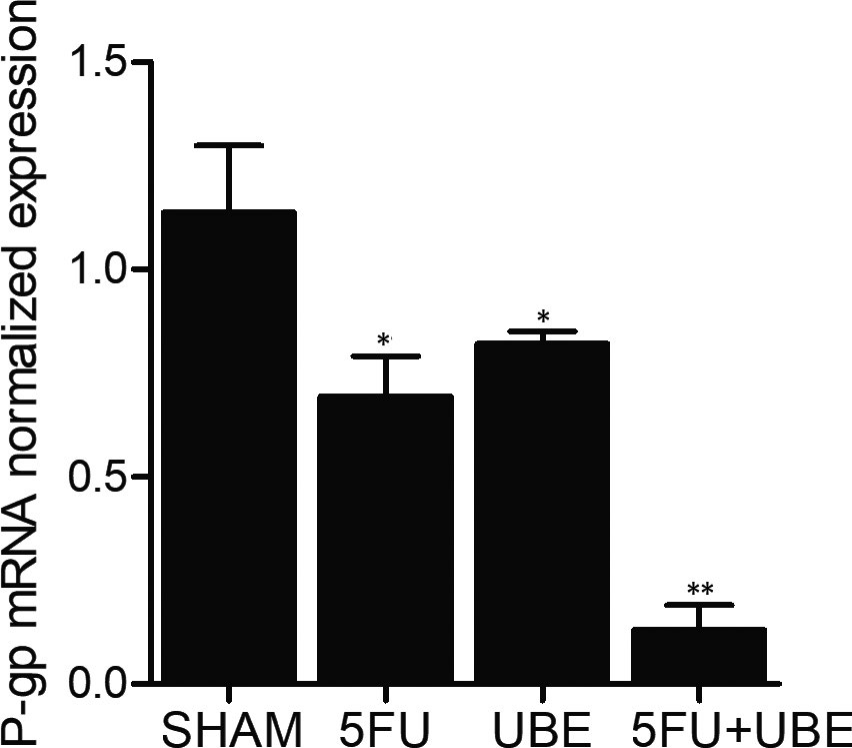
The expression of P‐gp mRNA in A549/ADR bearing mice was apparently suppressed by the combined administration of 5FU and UBE (*P < .05 compared with SHAM group; **P < .05 compared with 5FU group)

**FIGURE 3:**
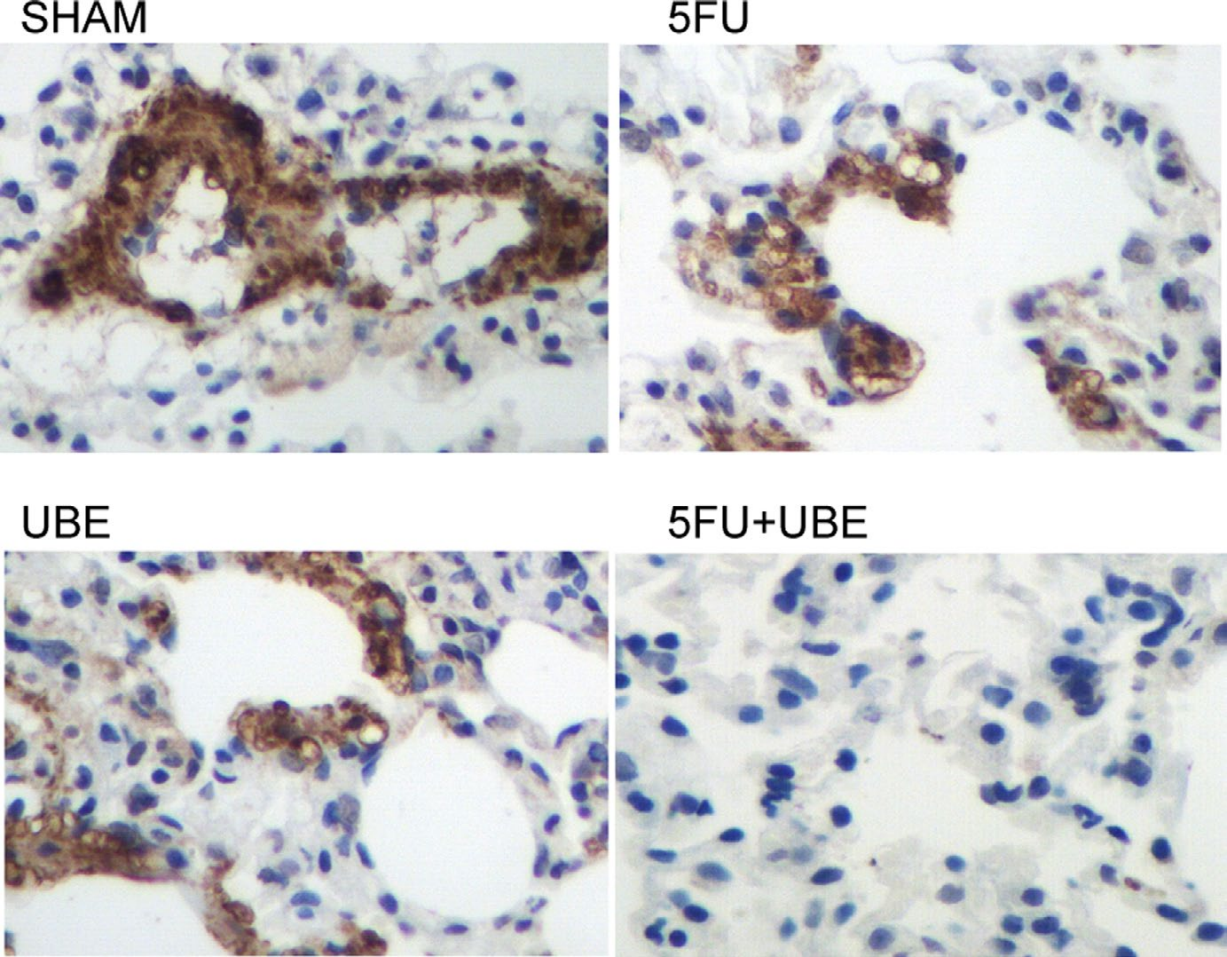
The expression of P‐gp protein in A549/ADR bearing mice was apparently suppressed by the combined administration of 5FU and UBE

Real‐time PCR was performed to evaluate the expression of P‐gp mRNA in the nude mice bearing A549/ADR tumour. Compared with 5FU and UBE mono‐therapies, the expression of P‐gp mRNA was more significantly repressed by the combined administration of 5FU and UBE in A549/ADR bearing nude mice (Figure 2). Moreover, immunohistochemistry was carried out to analyse the protein expression of P‐gp in the A549/ADR bearing nude mice treated with different strategies. Similar to the results of P‐gp mRNA expression, the protein expression of P‐gp was more significantly repressed by the combined administration of 5FU and UBE than 5FU and UBE mono‐therapies (Figure 3).

### 3.3 Combined administration of 5FU and UBE enhanced the apoptosis of A549/ADR

**FIGURE 4:**
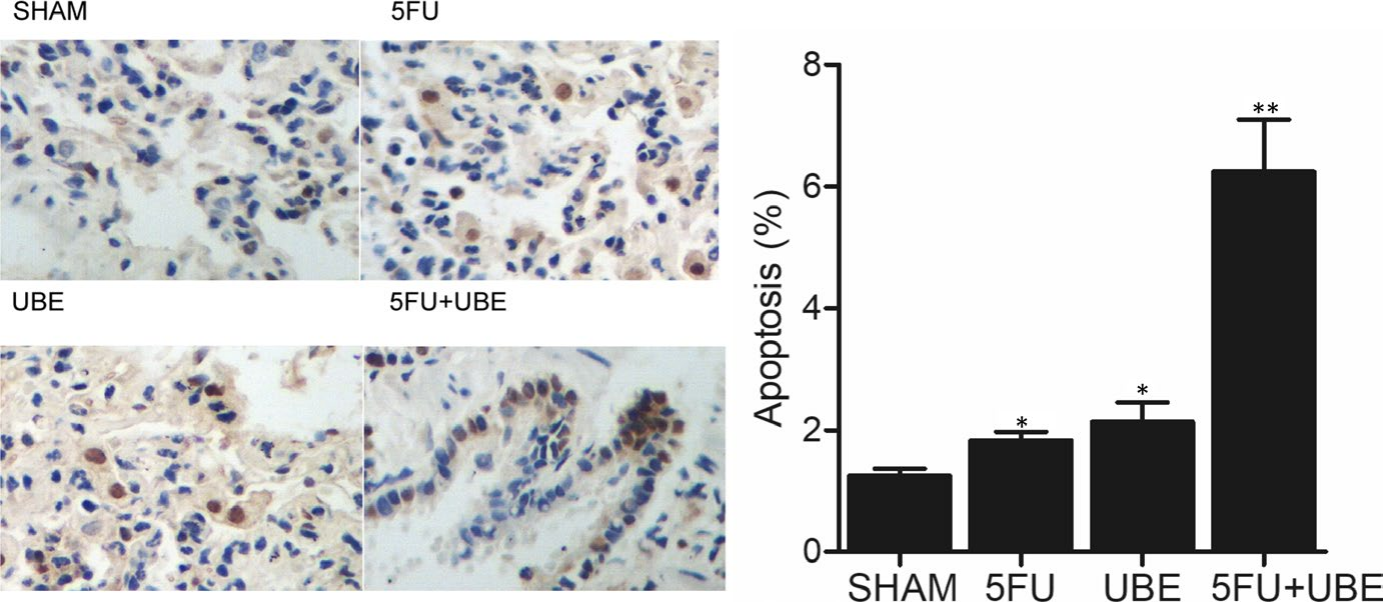
The apoptosis of cancer cells in A549/ADR bearing mice was notably elevated by the combined administration of 5FU and UBE (*P < .05 compared with SHAM group; **P < .05 compared with 5FU group)

Cell apoptosis is the most important criteria used to evaluate drug response. TUNEL assay was carried out here to analyse the apoptosis of A549/ADR cells in nude mice undergoing 5FU and UBE mono‐therapies or the combined administration of 5FU and UBE. Compared with 5FU and UBE mono‐therapies, the apoptosis of A549/ADR cells was more significantly enhanced by the combined administration of 5FU and UBE, indicating that the combined administration of 5FU and UBE could effectively improve the drug response of A549/ADR (Figure 4).

### 3.4 Combined administration of 5FU and UBE decreased the cell viability of A549/ADR

**FIGURE 5:**
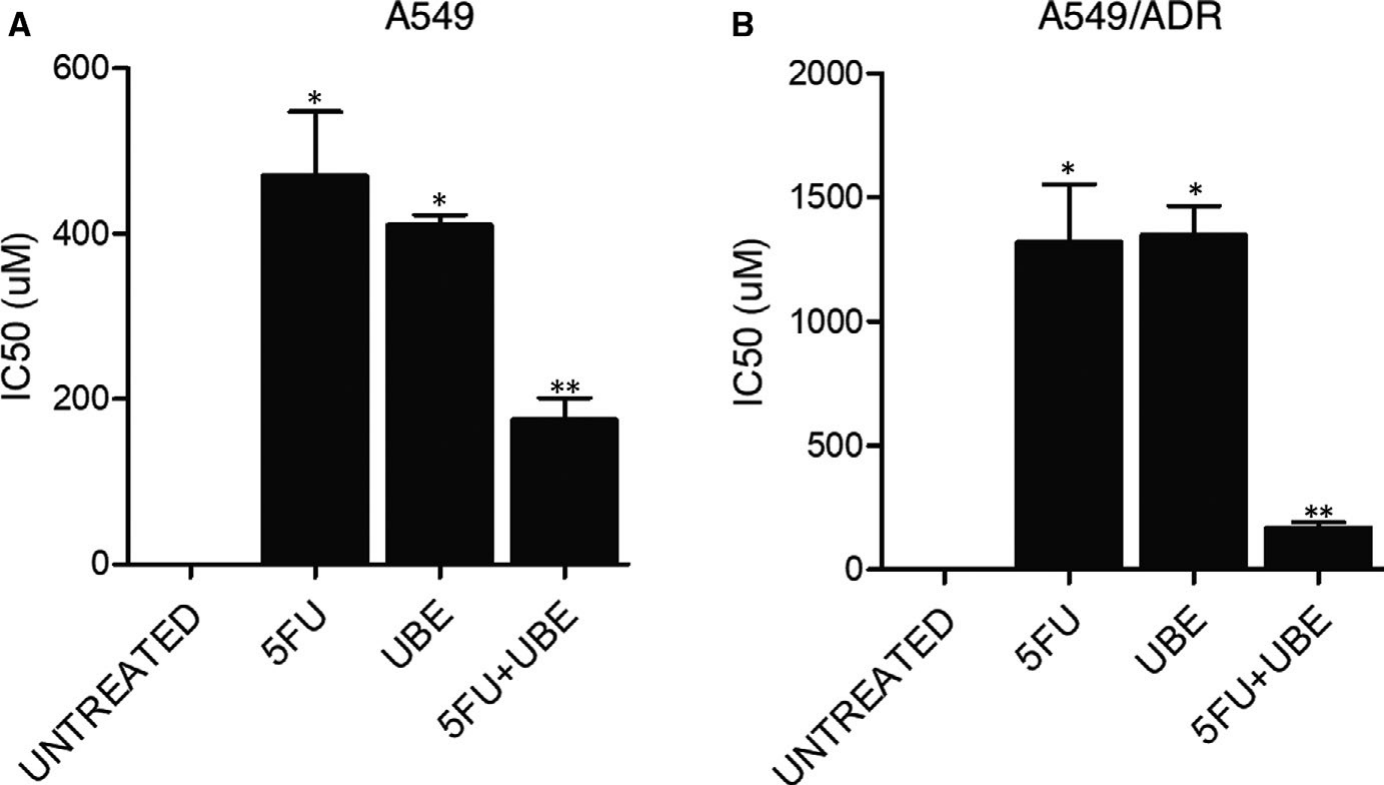
The cell viability of A549/ADR in nude mice was significantly inhibited by the combined administration of 5FU and UBE (*P < .05 compared with UNTREATED group; **P < .05 compared with 5FU group). A, The IC50 of 5FU + UBE in A549 bearing nude mice was lower than that of 5FU or UBE alone. B, The IC50 of 5FU + UBE in A549/ADR bearing nude mice was lower than that of 5FU or UBE alone

In order to further investigate the response of A549 and A549/ADR cells to 5FU and UBE, MTT assays were performed to determine the IC50 of 5FU and UBE in A549 and A549/ADR cells. The IC50 value of the combined administration of 5FU and UBE was much lower than that of 5FU and UBE mono‐therapies in A549/ADR cells, but the effect of the combined administration of 5FU and UBE was much more limited in A549 cells (Figure 5).

### 3.5 Treatment with 5FU and UBE enhanced the intracellular accumulation of the drugs in A549/ADR cells

**FIGURE 6:**
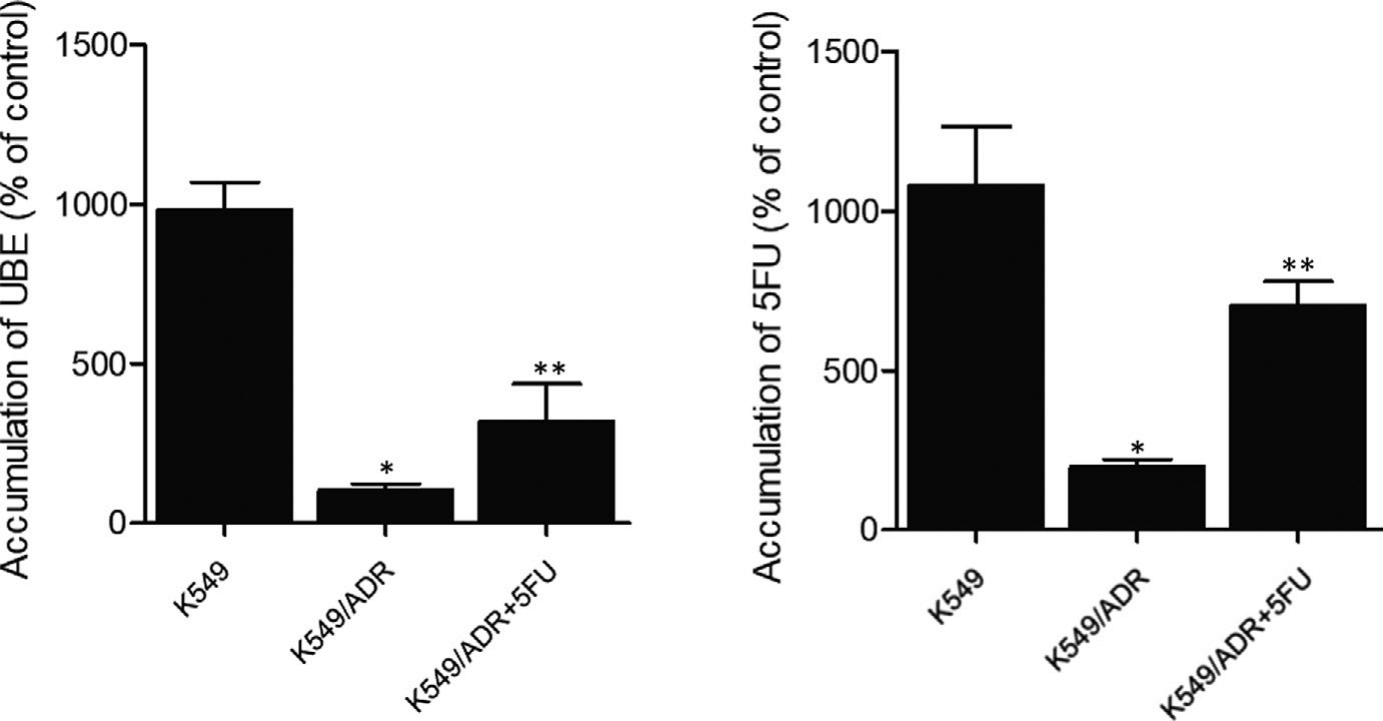
The intracellular accumulation of 5FU and UBE was enhanced in a synergistic manner by treating A549/ADR cells with 5FU + UBE (*P < .05 compared with A549 group; **P < .05 compared with A549/ADR group). A, Treatment of A549/ADR with 5FU improved the intracellular accumulation of UBE. B, Treatment of A549/ADR with UBE improved the intracellular accumulation of 5FU

As the intracellular accumulation of tumour killing drugs is a key index for the anti‐tumour activity of the drugs, we studied how the combination therapy of 5FU and UBE increased the drug response of A549/ADR by measuring the intracellular accumulation of 5FU and UBE in A549/ADR cells. As shown in Figure 6, the accumulation of UBE (Figure 6A) and 5FU (Figure 6B) was remarkably higher in A549 in comparison with that in A549/ADR. However, when A549/ADR cells were treated with the combined administration of 5FU and UBE, the accumulation of UBE was elevated by about 6 folds. On the other hand, the accumulation of 5FU in A549/ADR cells treated with the combined administration of 5FU and UBE was also enhanced significantly. These results indicated that combined administration of 5FU and UBE exerted a synergistic effect on their accumulation, leading to an increased level of anti‐tumour activity.

### 3.6 Combined administration of 5FU and UBE inhibited the expression of P‐gp in A549/ADR cells

**FIGURE 7:**
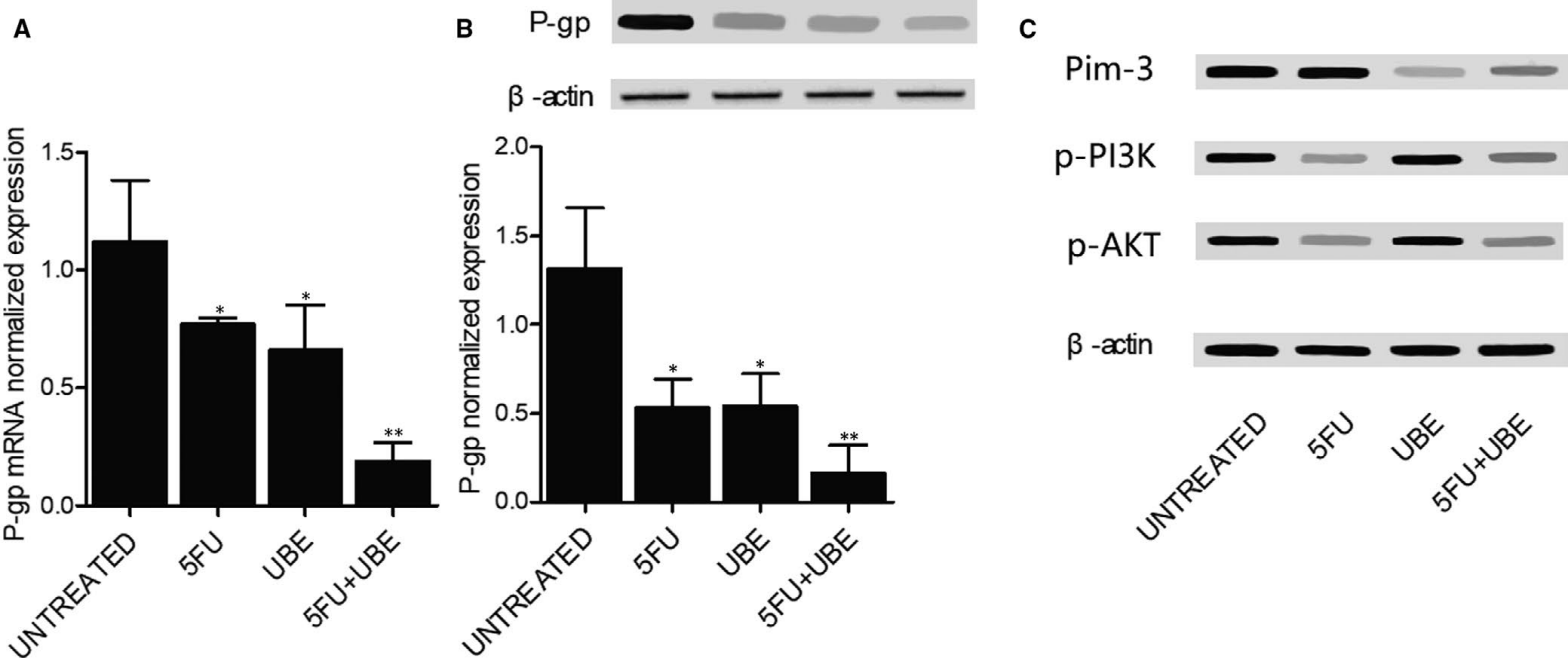
The expression of P‐gp in A549/ADR was apparently suppressed by the combined administration of 5FU and UBE. UBE treatment suppressed the expression of PIM‐3 while 5FU treatment down‐regulated the expression of p‐PI3K and p‐AKT (*P < .05 compared with UNTREATED group; **P < .05 compared with 5FU group). A, The expression of P‐gp mRNA was substantially attenuated in A549/ADR cells by the combined administration of 5FU and UBE. B, The expression of P‐gp protein was substantially attenuated in A549/ADR cells by the combined administration of 5FU and UBE. C, UBE treatment suppressed the expression of PIM‐3 while 5FU treatment down‐regulated the expression of p‐PI3K and p‐AKT

Real‐time PCR and Western blot were performed to analyse the mRNA and protein expression of P‐gp in A549/ADR cells treated with 5FU and UBE alone or with the combined administration of 5FU and UBE. As shown in Figure 7, when A549/ADR cells were treated with 5FU or UBE, the expression level of P‐gp mRNA (Figure 7A) and protein (Figure 7B) was evidently decreased. Moreover, upon the combined administration of 5FU and UBE, the P‐gp mRNA (Figure 7A) and protein expression was most significantly inhibited. Moreover, as shown in Figure 7C, UBE treatment other than 5FU inhibited PIM‐3 expression while 5FU treatment other than UBE treatment inhibited p‐PI3K and p‐AKT expression, which indicated that UBE and 5FU treatment respectively modulated the expression of P‐gp via regulating PIM‐3 and p‐PI3K/p‐AKT. The synergistic inhibitory effect of 5FU administration and UBE administration upon P‐gp mRNA and protein expression was therefore validated.

## 4 DISCUSSION

As a primary challenge faced by the treatment of various malignancies, MDR can be triggered by an elevated level of P‐gp expression. In cancer cells, P‐gp acts as a pump of drug efflux to effectively reduce the accumulation of chemotherapeutic drugs in the cells, thus increasing the anti‐cancer activity of these chemotherapeutic drugs. Indeed, cancer cells overexpressing P‐gp proteins are associated with a decreased activity of caspases, including caspase‐8 and caspase‐3, which are implicated in cell apoptosis.^11^, ^12^ In this study, we established a MDR cell line A549/ADR and carried out a study on tumour growth inhibition to assess the inhibitory effect of 5FU and UBE on tumour growth. We found that the combined administration of 5FU and UBE effectively overcame the MDR and promoted the drug response of MDR tumours in a mouse model.

The increased level of P‐gp expression can render cancer cells resistant to drugs with the ability to block cell mitosis. In addition, P‐gp acts as a membrane pump to pump out chemotherapeutic drugs to minimize their toxicity in cancer cells.^13^, ^14^, ^15^, ^16^ As a result, the understanding of the mechanisms underlying the desensitization of cancer cells overexpressing P‐gp against the toxicity of chemotherapeutic drugs may help us to develop better therapies for cancer patients showing MDR.^17^, ^18^ In this study, we evaluated the expression of P‐gp in A549/ADR tumour cells treated with 5FU and UBE alone or the combined administration of 5FU and UBE. The results showed that the combined administration of 5FU and UBE more significantly decreased the expression of P‐gp in A549/ADR.

In many patients of non‐small cell lung cancer, the development of MDR markedly reduces the efficacy of chemotherapy. Currently, the consensus is that the onset of MDR is attributed to three key proteins, that is topoisomerase‐II, P‐gp and glutathione S‐transferase pi. Among these three key proteins, transmembrane protein P‐gp has a molecular weight of 170 kDa and acts as a membrane pump to pump out chemotherapeutic drugs. In addition, the MDR1 gene encodes the P‐gp protein and the expression of MDR1 shows a negative correlation with the response and efficacy of chemotherapy.^19^ Moreover, the expression level of P‐gp proteins is markedly elevated in metastatic cancer cells, indicating that the increased level of P‐gp expression contributes to the development of cancer metastasis.^19^

As an immune‐modulator with anti‐tumour activity, UBE was shown to significantly inhibit the expression of P‐gp to promote the UBE absorption through the intestinal tract in rats.^20^

Bcl‐2, β3 tubulin and P‐gp can all increase the MDR of cancer cells against taxanes. For example, P‐gp acts as a transmembrane pump to pump out chemotherapeutic drugs including doxorubicin and taxanes from cancer cells. In addition, the function of P‐gp can be significantly suppressed by 5FU.

The level of expression of P‐gp in SGC 7901 cells can be significantly decreased by treating the cells with 5FU. On the other hand, the maintenance in the level of P‐gp expression is crucial for the survival of cancer cells.^21^, ^22^ For example, when a MDR clone of SGC 7901 cells was established, its level of expression of P‐gp was found to be significantly increased. In this study, we used a TUNEL assay to evaluate the apoptosis of A549/ADR tumour cells treated by 5FU and UBE alone or the combined administration of 5FU and UBE. The results showed that the combined administration of 5FU and UBE more remarkably enhanced the apoptosis of A549/ADR cells by enhancing the intracellular accumulation of the drugs in a synergistic manner. Furthermore, we carried out MTT assays to calculate the IC50 value of 5FU and UBE alone or the combined administration of 5FU and UBE in A549/ADR cells. The results showed that the IC50 value of the combined treatment of 5FU and UBE in A549/ADR was much lower than that of 5FU and UBE alone. UBE can also reduce the levels of BCL‐XL and BCL‐2 expression in HepG2 cells. In addition, UBE can significantly decrease the expression level of phosphorylated Bad without affecting the overall level of Bad proteins. Therefore, the cytotoxic effect of UBE may be mediated through its promotion of apoptosis.

There are limitations in this study. Since only the data from cell lines and animal model is available, further studies in NSCLS patients treated with 5FU, bestatin or the combination therapy are warranted.

## 5 CONCLUSION

To sum up, the results of this study demonstrated that UBE combined with fluorouracil attenuated MDR and inhibited the expression of P‐gp in lung cancer.

## CONFLICT OF INTEREST

None.

## AUTHOR CONTRIBUTION

**Jun Wan:** Conceptualization (equal); Formal analysis (equal); Investigation (equal); Methodology (equal); Supervision (equal); Writing‐original draft (equal). **Xie‐an Ling:** Investigation (equal); Methodology (equal); Validation (equal); Writing‐original draft (equal). **Jian Wang:** Investigation (equal); Resources (equal); Software (equal). **Guang‐gui Ding:** Investigation (equal); Methodology (equal); Validation (equal); Visualization (equal). **Xi Wang:** Conceptualization (equal); Data curation (equal); Formal analysis (equal); Project administration (equal); Writing‐review & editing (equal).

## Data Availability

Please contact the corresponding author for request for the datasets used and/or analysed in this study.
